# Early elective versus elective sigmoid resection in diverticular disease: not only timing matters—a single institutional retrospective review of 133 patients

**DOI:** 10.1007/s00423-022-02464-1

**Published:** 2022-02-22

**Authors:** Sascha Vaghiri, David Mario Jagalla, Dimitrios Prassas, Wolfram Trudo Knoefel, Andreas Krieg

**Affiliations:** Department of Surgery (A), Heinrich-Heine-University and University Hospital Duesseldorf, Moorenstr. 5, Bldg. 12.46, 40225 Duesseldorf, Germany

**Keywords:** Diverticular disease, Classification of diverticular disease, CDD, Laparoscopic surgery, Timing of surgery

## Abstract

**Purpose:**

The optimal timing of elective surgery in patients with the colonic diverticular disease remains controversial. We aimed to analyze the timing of sigmoidectomy in patients with diverticular disease and its influence on postoperative course with respect to the classification of diverticular disease (CDD).

**Methods:**

Patients who underwent elective laparoscopic sigmoidectomy were retrospectively enrolled and subdivided into two groups based on the time interval between the last attack and surgery: group A, early elective (≤ 6 weeks), and group B, elective (> 6 weeks). Multivariate regression models were used to identify factors which predict conversion to laparotomy, postoperative course, and length of hospital stay.

**Results:**

A total of 133 patients (group A (*n* = 88), group B (*n* = 45)) were included. Basic demographic data did not differ between groups except for a higher rate of diabetes in group B (*p* = 0.009). The conversion rate was significantly higher in group A in comparison to group B (group A vs. group B: *n* = 23 (26.1%) vs. *n* = 3 (6.7%), *p* = 0.007). Logistic regression analysis revealed the timing of surgery and CDD stage as significant predictors for intraoperative conversion. Moreover, the postoperative course was influenced by high age as well as intraoperative conversion and length of hospital stay by conversion, preoperative CRP levels, and elective surgery.

**Conclusions:**

Both, timing of surgery and the disease stage, influence the conversion rates in laparoscopic sigmoidectomy for diverticular disease. Accordingly, patients with complicated acute or chronic sigmoid diverticulitis should be operated in the inflammation-free interval.

**Supplementary Information:**

The online version contains supplementary material available at 10.1007/s00423-022-02464-1.

## Introduction

Diverticular disease of the colon has a steadily increasing prevalence in western countries. While the prevalence is about 5% among patients aged 40 or younger, it affects more than 50% of the patients above the age of 60 years [1]. This epidemiologic observation has not only serious impact on clinical management but also reveals a striking socioeconomic burden in our healthcare system. In Germany alone, more than 130,000 patients were hospitalized due to diverticular disease according to official federal data from 2016 [2]. New pathophysiological insights and evolved understanding of sigmoid diverticular disease over the past decades have led to a shift in the treatment strategies towards disease stage-adapted and patient-tailored approaches abandoning old paradigms [3, 4]. The therapeutical option for uncomplicated stages of the acute diverticular disease consists of a medical-conservative management given its low risk of disease progression and recurrence [2, 5, 6]. However, in patients with certain risk factors (e.g., immunosuppression) an elective surgical resection after rehabilitation should be considered [4, 7]. Patients suffering from an acute complicated diverticular disease with abscess formation should initially be considered for conservative medical management including antibiotic therapy and abscess drainage during the acute inflammatory period. After cessation of the acute episode, an elective resection is recommended owing to high recurrence and related morbidity and mortality rates [2–4, 8]. Free perforation or failed conservative treatment of acute complicated diverticulitis mandates immediate surgical intervention [2, 3, 9]. For patients with chronic recurrent disease, the therapeutic regimen ranges from outpatient conservative to surgical therapy. Especially for those suffering from recurrent types complicated by fistula, stenosis, or conglomerate masses, laparoscopic sigmoid resection should be considered. However, the optimal timing of laparoscopic sigmoid resection in diverticular disease is still a matter of debate with inconsistent recommendations throughout the European and American societies due to missing randomized controlled trials (RCT) highlighting this question [2, 7, 10]. A recently published meta-analysis favors delayed elective resection with shorter operative and hospitalization times and lower conversion rates compared to the early elective approach, although no significant procedure-related differences in outcome could be demonstrated [11].

In this study, we conducted a retrospective single-institutional analysis of all patients electively treated by laparoscopic sigmoid resection for acute or chronic sigmoid diverticular disease at our department. Special focus was pointed towards the timing of elective resection in relation to the onset of symptoms and its impact on the perioperative outcome providing a reliable tool for individual decision-making in the setting of an acute inflammatory attack or chronic disease burden.

## Material and methods

### Study collective and perioperative management

All patients with an acute or chronic sigmoid diverticular disease undergoing laparoscopic sigmoidectomy at our department from January 2004 to July 2021 were retrospectively analyzed. Relevant data on demographics, comorbidities, recent attacks of diverticulitis, clinical and radiological features at the time of presentation, abscess-drainage placement, intraoperative, and postoperative course, including morbidity and mortality after sigmoidectomy, were extracted from our prospectively maintained medical database. These data included age, gender, body mass index (BMI), American Society of Anesthesiologists (ASA) score, relevant comorbidities (e.g., cardiovascular or metabolic diseases) and immunosuppression, preoperative laboratory parameters including inflammatory markers and hemoglobin, preoperative medical course, time since the last attack to surgery, type and duration of surgery, conversion rate, necessity of an ostomy creation, intraoperative transfusion, all documented postoperative minor and major complications until the date of hospital discharge, and the total hospitalization time. The postoperative morbidities were categorized according to the Clavien-Dindo classification [12]. A critical review of clinical, radiographic, and intraoperative data enabled an accurate classification of the disease stage in each patient. This was categorized using the classification of diverticular disease (CDD) [6]. In addition, we divided our collective into 2 separate groups depending on the time of surgery: group A represents the early elective cohort with patients who underwent a laparoscopic sigmoidectomy within 6 weeks of the last attack of acute or complicated diverticular disease. Group B comprises patients who received surgical therapy in the non-inflammatory interval at least 6 weeks after the last attack and documented relief of symptoms associated with diverticular disease (elective group). Patients representing with large intraabdominal abscess formation were initially considered for interventional drainage placement. Our standard operation procedure (S.O.P) for early elective and elective sigmoidectomy was as follows: Upon presentation in our department, every patient with the suspected diverticular disease received after clinical examination abdominal computed tomography (CT) imaging beside clinical and laboratory tests. Cases with free perforation (CDD 2c) were directly scheduled for emergency surgery. Patients with acute or chronic symptomatic diverticular disease were admitted. Based on the imaging results, larger mesocolic or pelvic abscess formations were drained interventionally if applicable. Our standard i.v. antibiotic regimen included ceftriaxon 2 g (once daily) and metronidazol 500 mg (thrice daily) or in severe cases piperacillin 4 g/tazobactam 0.5 g every 6 h for at least 5–7 days beside initial parenteral feeding and gradual oral nutrition intake in the latter course. Patients who responded to the conservative treatment by means of complete symptom relief and normalizing laboratory parameters were either discharged and referred to our outpatient clinic for a close follow-up or given the opportunity for an early elective resection based on their personal preference and quality of life burden in close interaction with the responsible surgeon. Thus, the decision towards early elective and elective resection was based on the surgeons’ personal experience and preference alongside the clinical presentation and success of the medical therapy taking into account the current German guidelines of diverticular disease [6] and each patient individual decision as well as quality of life expectations and restrictions. However, patients with persistent symptoms, apparent clinical deterioration despite medical therapy, or known risk factors (e.g., immunosuppression or relevant comorbidities) for a more complicated disease course underwent an early elective resection during index hospitalization although not initially intended. The surgical strategy did not differ between the early elective and the elective cases in our study. Note that we excluded patients with free perforation (CDD type 2c) or sepsis requiring emergency surgery, as well as patients with primarily open procedures from our analysis. Only specialized surgeons (board-certified visceral surgeons according to the requirements of the Federal German Medical Council) performed the procedures. If necessary, especially in the case of decision-making for conversion to laparotomy, a senior consultant or the head of the department was involved during the operation. All surgeons were trained and skilled in open and laparoscopic colorectal surgery. Each surgeons’ personal surgical statistics are recorded in our database. Briefly, after placement of 4 ports, lateral to medial mobilization, splenic flexure mobilization and low tie ligation were usually performed. The specimen was removed through a Pfannenstiel incision of approximately 5 cm in length. Restoration of the gastrointestinal passage was achieved through an end-to-end anastomosis using a 31-mm circular stapler. Intraoperative findings and the surgeon’s decision determined the operative course including conversion via median laparotomy and ostomy creation. Standard postoperative care with routine laboratory evaluation, mobilization, and gradual return to a normal diet after a documented bowel movement was administered. Patients with postoperative prolonged bowel paralysis were put on additional total parenteral nutrition (TPN). After discharge from the hospital, all patients were seen on regular basis for clinical follow-up at our department.

### Ethical approval

An ethical approval was obtained prior to the study commencement by the institutional ethical board of the Medical Faculty, Heinrich-Heine University Duesseldorf, Germany (study-no.: 2021–1346) and all reported procedures and steps were in accordance with the current ethical standards and requirements in the latest version of the Declaration of Helsinki.

### Statistical analysis

Statistical analysis was performed using G*Power [13], SPSS 23.0 software (IBM Corp., Armonk, NY), and R version 4.1.1 with packages MICE [14], Hmisc, readxl, rms, ResourceSelection, and MASS. The normal distribution of continuous variables was assessed by the Kolmogorov–Smirnov and Anderson–Darling test. For descriptive data analysis, continuous variables were compared using the Mann–Whitney *U* or *t*-test. Categorical variables were compared using Fisher’s exact or chi-square test. Post hoc statistical power regarding the primary outcomes of the study, conversion rate depending on the timing of surgery or CDD, was found to be 80.9% and 98.5% respectively, assuming a significance level of 0.05. For missing data, we used the MICE package which imputes incomplete multivariate data by chained equations. The binary logistic regression model was used when the dependent variable had two categories. For this purpose, continuous variables remained unchanged when normally distributed (age) or were transformed by grouping either according to clinically relevant classifications (BMI, CDD), median (operation time), or by using the log_10_ function (CRP, leucocytes). To establish a clinical selection model, we first screened for independent variables based on a stepwise backward selection in which the model with the lowest Akaike information criterion (AIC) is selected. Next, for cross-validation, we used the bootstrap method by resampling 100 times with replacement to assess the consistency of the predictors selected with the stepwise selection. Finally, we performed the logistic regression model that was suggested from the bootstrap method. The independent influencing factors were utilized to draw a nomogram. Model discrimination ability was assessed by C-statistics. A C-index of 1 indicates that the model perfectly predicts the outcome, over 0.8 and over 0.7 indicates a strong and good model, respectively, and a value of 0.5 means that the model is not predicting better than random chance. The goodness-of-fit (GOF) was evaluated by the Hosmer–Lemeshow test in which *p*-values < 0.05 indicate poor fit. Due to the small sample size, we validated the reproducibility of our model internally by the bootstrap method by resampling 100 times and assessing calibration curves. A linear regression model was performed using stepwise backward selection when there was a dependent continuous variable. In all analyses, a *p*-value of < 0.05 indicated statistical significance.

## Results

### Patient characteristics

During the study period, a total of 133 patients underwent laparoscopic sigmoid colon resection due to diverticular disease at our department. Based on the cutoff value of 6 weeks since the onset of symptoms and the surgical procedure, 88 patients (66.2%) were enrolled in the early elective group A and 45 patients (33.8%) in the elective group B. Patient characteristics are summarized in Table 1. There were 69 men (51.9%) and 64 women (48.1%) with a median age of 55 years at the time of surgery. Baseline demographic data including sex, age, BMI, and ASA score did not differ between the two groups. Comorbidities were equally distributed apart from the incidence of diabetes which was significantly lower in the early elective group (*p* = 0.009). A review of the preoperative laboratory parameters revealed significantly higher inflammatory values (white blood cell, WBC count, and C-reactive protein, CRP) in group A vs. group B (*p* < 0.001) as well as decreased sodium levels (*p* = 0.003) prior to surgery. Patients in both groups suffered from a median of two diverticulitis attacks before surgery (*p* = 0.843). In the early elective group, 7 patients (8%) underwent abscess drainage in comparison to 2 patients (4.4%) in the elective group (*p* = 0.446). The median time interval between the last episode of diverticulitis and sigmoid resection was significantly shorter with 12 days (± 10.2 days) in group A vs. 62 days (± 35.5 days) in group B (*p* < 0.001). In both groups, an equal distribution of diverticulitis stages according to CDD was observed (*p* = 0.304).

**Table 1:** Patient characteristics

	All patients *n* = 133	Group A (early elective) *n* = 88	Group B (elective) *n* = 45	*P*-value
Sex (***n***; %)				
Male	69 (51.9)	42 (47.7)	27 (60)	0.180
Female	64 (48.1)	46 (52.3)	18 (40)	
Age (median ± SD)	55 ± 12.3	56 ± 12.3	53 ± 13.1	0.602
BMI (median ± SD)	26.6 ± 4.9	26.6 ± 5.4	25.9 ± 3.9	0.545
ASA score (***n***; %)				
I	25 (18.8)	12 (13.6)	13 (28.9)	0.160
II	59 (44.4)	38 (43.2)	21 (46.7)	
III	18 (13.5)	14 (15.9)	4 (8.9)	
IV	3 (2.3)	1 (1.1)	2 (4.4)	
NA	28 (21.1)	23 (26.1)	5 (11.1)	
Comorbidities (***n***; %)				
Hypothyreosis	22 (16.5)	14 (15.9)	8 (17.8)	0.752
Diabetes	6 (4.5)	1 (1.1)	5 (11.1)	0.009
Arterial hypertension	46 (34.6)	28 (31.8)	18 (40.0)	0.372
Chronic renal insufficiency	8 (6)	6 (6.8)	2 (4.4)	0.576
Immunosuppression	6 (4.5)	5 (5.7)	1 (2.2)	0.357
Laboratory data (preop.)				
Na (mmol/l) [median ± SD]	140 ± 3.3	139.5 ± 3.5	141 ± 2.5	0.003
CRP (mg/dl) [median ± SD]	2.2 ± 7.2	4.6 ± 7.6	0.3 ± 3.6	0.000
WBC (× 1000/μl) [median ± SD]	8.7 ± 4.8	10.7 ± 5.2	7.5 ± 2.6	0.000
Hb (g/dl) [median ± SD]	13.9 ± 2.2	13.7 ± 2.2	14.4 ± 2.2	0.168
Thrombocytes (× 1000/μl) [median ± SD]	261.5 ± 138.1	264 ± 159.9	264 ± 76.1	0.943
Number of attacks prior to surgery (median ± SD)	2 ± 1.4	2 ± 1.2	2 ± 1.6	0.843
CT drainage prior to surgery (***n***; %)	9 (6.8)	7 (8.0)	2 (4.4)	0.446
Interval between CT-drainage and surgery (days, median ± SD)	21 ± 28.1	18 ± 6.7	72 ± 33.9	0.040
Interval between last attack and surgery (days, median ± SD)	20 ± 37.4	12 ± 10.2	62 ± 35.5	0.000
CDD classification (***n***; %)				
1b	12 (9.0)	8 (9.1)	4 (8.9)	0.304
2a	37 (27.8)	25 (28.4)	12 (26.7)	
2b	22 (16.5)	19 (21.6)	3 (6.7)	
3a	2 15)	1 (1.1)	1 (2.2)	
3b	35(26.3)	21 (23.9)	14 (31.1)	
3c	25 18.8	14 (15.9)	11 (24.4)	

*BMI*, body mass index; *ASA score*, American Society of Anesthesiologists; *CRP*, C-reactive protein; *WBC*, white blood cells

### Surgical data

**Fig. 1:**
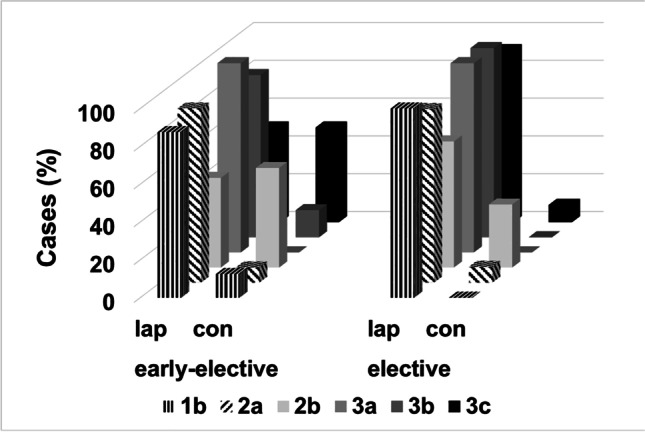
Distribution of different types of diverticular disease according to the CDD in the early elective and elective surgery group (lap = laparoscopic, con = conventional)

The median operation time was comparable within both groups (group A: 295 ± 65.5 min vs. group B: 279 ± 84.3 min; *p* = 0.553). In group A sigmoid resection with a primary anastomosis was performed in 94.3% (*n* = 83) and in 4.5% (*n* = 4), a protective diversion ostomy was additionally constructed. A Hartmann procedure was necessary for one patient (1.1%). In contrast, all patients in group B underwent primary resection with anastomosis (100%) and without an ostomy. Regarding the need for intraoperative transfusion of blood units, crystalloid or colloidal infusions no significant differences between the groups became evident. A summary of available operative data is depicted in Table 2. However, conversion rates to laparotomy were significantly higher with 26.1% (23 of 88 patients) in group A as opposed to only 6.7% (3 of 44 patients) in group B (*p* = 0.007). The main reasons for conversion were severe inflammatory adhesions (*n* = 17), inflammatory conglomerate (*n* = 6), or uncontrollable bleeding (*n* = 3). To further elucidate a possible relationship between disease stage and conversion rate, we graphically displayed conversion rates depending on the CDD for the entire study group (Supplementary Fig. 1). Accordingly, in patients undergoing laparoscopy for diverticular disease, we observed higher conversion rates to laparotomy for type 2b (50%) and 3c (32%) disease. Next, we analyzed the disease stage-dependent conversion rates according to the time point of surgery. Interestingly, conversion rates were only significantly higher (*p* = 0.038) in group A patients suffering from CDD 3c when compared to patients having elective surgery for diverticular disease (Fig. 1). Of note, in the group of patients with CDD type 2b who were operated in the early elective period, the majority (14 out of 19 patients) demonstrated a complete resolution of the acute attack after medical treatment and preferred early elective surgery after information about the therapeutic strategies (early elective vs. elective surgery). Out of the 14 patients with positive medical response, conversion to laparotomy was necessary for 7 patients (50%). Of the remaining patients with CDD type 2b diverticulitis refractory to conservative treatment who underwent early elective surgery (*n* = 5), conversion to laparotomy was performed in 3 cases. Although statistical analysis showed no significance when comparing the conversion rate with the response rate (*p* = 0.56), due to the small number of cases, these observations should be interpreted with caution.

**Table 2:** Surgical data

	All patients *n* = 133	Group A (early elective) *n* = 88	Group B (elective) *n* = 45	*P-*value
Duration of surgery (min) [median ± SD]	296.9 ± 71.8	279 ± 65.5	295 ± 84.3	0.553
Type of surgery (***n***; %)				
Primary laparoscopic sigmoid resection	107 (80.5)	65 (73.9)	42 (93.3)	0.007
Conversion to open laparotomy	26 (19.5)	23 (26.1)	3 (6.7)	
Sigmoid resection with primary anastomosis	128 (96.2)	83 (94.3)	45 (100)	0.103
Sigmoid resection with primary anastomosis and protective ostomy (***n***; %)	4 (3)	4 (4.5)	0 (0)	0.146
Hartman procedure (***n***; %)	1 (0.8)	1 (1.1)	0 (0)	0.473
Type of protective ostomy (***n***; %)				
Ileostomy	2 (1.5)	2 (2.3)	0 (0)	0.550
Tranversostomy	2 (1.5)	2 (2.3)	0 (0)	
Intraoperative transfusion				
Cristalloid infusion (ml) [median ± SD]	5000 ± 2206	5000 ± 2207	5000 ± 2225	0.627
Blood units (ml) [median ± SD]	0 ± 201.3	0 ± 197.9	0 ± 209.9	0.920
Fresh frozen plasma (ml) [median ± SD]	0 ± 178	0 ± 221.1	0 ± 0	0.293

The most common inflammatory manifestation of the chronic complicated diverticulitis in the CDD type 3c subgroup was a luminal obstruction (early elective *n* = 11 vs. elective *n* = 9). However, none of these patients experienced an acute mechanical bowel obstruction requiring emergent surgical intervention. One patient with recurrent diverticulitis and a colo-vesical fistula underwent early resection. In each group, 2 patients with concomitant luminal stenosis and conglomerate mass were included. The reasons for an early elective approach in the CDD type 3c subgroup (*n* = 14) were as follows: colo-vesical fistula with bacteremia (*n* = 1), clinical deterioration with aggravating abdominal symptoms and subtotal stenosis (*n* = 3), and the remaining 10 patients displayed a positive response to the medical therapy and preferred an earlier surgical intervention. Among them, 2 patients experienced a history of ≥ 6 episodes of a diverticulitis of whom one patient had immunosuppressive medication after kidney transplantation. Three patients were aged 50 or younger at the time of early elective resection in the CDD type 3c subgroup after complete recovery from the acute attack. Note, in the early elective surgery group of patients with CDD type 3c, a conversion to laparotomy was indicated in 4 patients (40%) with a complete relief from symptoms and in 3 patients (75%) with persistent or increasing clinical symptoms (*p* = 0.28).

**Fig. 2:**
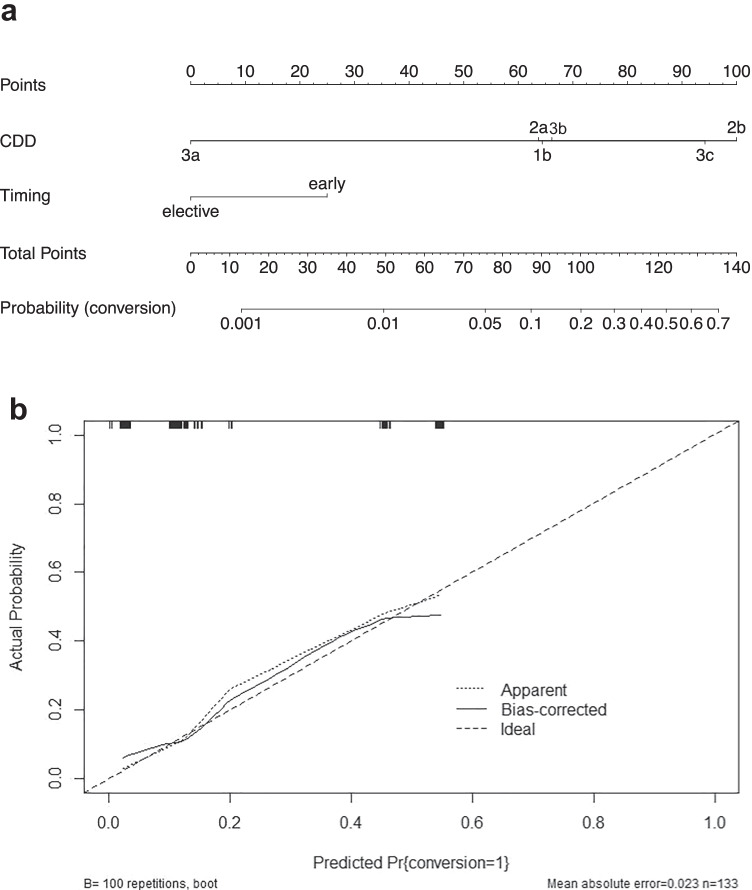
Nomogram for predicting the probability of conversion to laparotomy in patients undergoing laparoscopic sigmoid resection for diverticular disease. Nomogram (**a**) was constructed by multivariate regression analysis and calibration curve (**b**) revealed a well-calibrated model

Thus, our data gave a first indication that both the CDD stage and the time of surgery may predict surgical conversion rates. To test this hypothesis, we performed binary logistic regression analysis. Therefore, we included variables that might be clinically relevant such as age, sex, BMI, preoperative WBC and CRP, and CDD stage and timing of operation and conducted independent variable selection by applying a stepwise backward selection and bootstrap resampling. Using this approach, we identified CDD and timing of surgery as two factors that were associated with surgical conversion. Both covariates were entered in a final regression model and a nomogram was constructed (Table 3, Fig. 2a) in which from both predictors a vertical line can be drawn to the points axis. The points obtained by this way are added up to give the total points. Finally, a vertical line can be drawn from the total point axis to the “probability (conversion)” axis, which reflects the probability of conversion to laparotomy. For example, a patient undergoing early elective laparoscopic sigmoid resection (25 points) for CDD 2b (100 points) achieves a total number of 125 points which corresponds to a probability of approximately 55% for intraoperative conversion to laparotomy. Of note, a C-index of 0.798 indicated a good model. In addition, the Hosmer–Lemeshow test confirmed the GOF (*p* = 0.927) and the calibration curve was almost congruent with the ideal line, indicating a well-calibrated model (Fig. 2b).

**Table 3:** Multivariate logistic regression analysis of predictors for conversion to laparotomy

Selected variables	OR	95% CI	*P-*value
CDD			
1b	Reference		
2a	0.960	0.080–10.467	0.973
2b	9.562	1.016–89.995	0.048
3a	0.017	0.000–inf	0.837
3b	1.119	0.102–10.225	0.927
3c	6.625	0.688–63.843	0.102
Time of surgery			
Group A (early elective)	Reference		
Group B (elective)	0.205	0.0529–0.796	0.022

### Postoperative outcome

An uneventful postoperative course was recorded in 91 patients (68.4%). When comparing the rate of complications in group A and group B patients according to Clavien-Dindo [12], no statistically significant difference became evident (*p* = 0.540) (Table 4).

**Table 4:** Postoperative morbidity and mortality

	All patients *n* = 133	Group A (early elective) *n* = 88	Group B (elective) *n* = 45	*P*-value
Postoperative morbidity (***n***; %)				
Wound infection	26 (19.5)	17 (19.3)	9 (20)	0.925
Anastomotic insufficiency	5 (3.8)	4 (4.5)	1 (2.2)	0.505
Ileus (paralytic/mechanic)	1 (0.8)	1 (1.1)	0 (0)	0.473
Other morbidities (***n***; %)				
Bowel ischaemia	1 (0.8)	0 (0)	1 (2.2)	0.239
Urinary leakage	2 (1.5)	2 (2.3)	0 (0)	
Incisional hernia	1 (0.8)	1 (1.1)	0 (0)	
Intraabdominal abscess	1 (0.8)	1 (1.1)	0 (0)	
Clavien-Dindo classification (***n***; %)				
I	2 (1.5)	1 (1.1)	1 (2.2)	0.540
II	8 (6.0)	6 (6.8)	2 (4.4)	
IIIa	16 (12)	9 (10.2)	7 (15.6)	
IIIb	13 (9.8)	11 (12.5)	2 (4.4)	
IVa	2 (1.5)	2 (2.3)	0 (0)	
V	1 (0.8)	1 (1.1)	0 (0)	
Revisional surgery (***n***; %)	15 (11.3)	11 (12.5)	4 (8.9)	0.388
Duration of hospital stay after surgery (days, median ± SD)	9 ± 10.1	9 ± 8.4	9 ± 12.8	0.964

To identify predictors for an eventful postoperative course, which we defined as at least grade 1 complication according to the Clavien-Dindo classification, we again performed binary logistic regression analysis. The following variables were entered into a binary regression model performing a stepwise backward selection: Age, sex, WBC count, CRP, conversion to laparotomy, operation time, CDD, and time of surgery. Using bootstrap resampling, logistic regression analysis identified conversion to laparotomy and higher age as predictive variables for an eventful postoperative course (Table 5). Again, based on these results, we designed a nomogram to predict the probability of an eventful postoperative course (Supplementary Fig. 2a). The Hosmer–Lemeshow test (*p* = 0.339) as well as the calibration curve (Supplementary Fig. 2b) underlined the goodness of fit.

**Table 5:** Multivariate logistic regression analysis of predictors for an eventful postoperative course

Selected variables	OR	95% CI	*P-*value
Age	1.034	1.003–1.068	0.034
Conversion			
No	Reference		
Yes	2.612	1.066–6.400	0.036

Next, we were interested in identifying factors which affect hospital stay in patients who underwent laparoscopic surgery for diverticular disease. Hence, we initiated a backward stepwise linear regression by including age, sex, BMI, WBC count, CRP, timing of operation, operation time, conversion to laparotomy, and the postoperative course. Interestingly, our model found elevated preoperative CRP, conversion to open surgery, and an electively planned surgery to influence the length of hospital stay (Table 6). For example, a patient with a preoperative CRP of 1 mg/dl, receiving elective laparoscopic sigmoid resection without conversion and having an uneventful postoperative course will most likely be discharged on postoperative day 9. In contrast, for a patient with a preoperative CRP of 15 mg/dl who receives early elective laparoscopic surgery, but needs to be converted to laparotomy and experiences a complicated postoperative course, a hospital stay of 26 days was predicted.

**Table 6:** Multivariate linear regression analysis of predictors of hospital stay

Selected variables	Coefficient (*B*)	SE	*P*-value
Constant	13.5282	1.9356	< 0.001
CRP	0.3702	0.1198	0.003
Time of surgery			
Group A (early elective)	Reference		
Group B (elective)	4.2911	1.8377	0.021
Conversion			
No	Reference		
Yes	6.9160	2.0342	< 0.001
Postoperative course			
Eventful	Reference		
Uneventful	-9.3354	1.7012	< 0.001

## Discussion

The results of our retrospective study including 133 patients undergoing laparoscopic sigmoidectomy demonstrated significantly higher conversion rates in CDD 2b and 3c stages according to irrespective of surgical timing. However, subgroup analysis according to the time point of resection revealed higher conversion rates for CDD 3c in patients undergoing early elective sigmoid resection. After logistic regression analysis, timing of surgery along with CDD stages was identified as predictors of conversion. Moreover, we demonstrated that conversion to laparotomy together with higher age negatively influenced the postoperative course after laparoscopic sigma resection. In this context, we show that elective surgery, preoperatively elevated CRP, and conversion to laparotomy influenced the duration of postoperative stay.

The CDD classification was first introduced in 2014 in Germany [15, 16] as an instrument designed to assist clinicians in the process of therapeutic decision-making. The advantage of CDD over the established modified Hinchey classification relies on its ability to discriminate cases of acute complicated and recurrent diverticular disease [17, 18]. Acute complicated cases are described as covered perforation with micro-abscess ≤ 1 cm (type 2a) or macro-abscess > 1 cm (type 2b) and free perforation (type 2c). As a matter of fact, CDD is the only classification that subdivides cases with the chronic diverticular disease into three further subgroups. Subgroup CDD 3a refers to patients with symptomatic uncomplicated disease, whereas CDD 3b includes cases with relapsing uncomplicated diverticulitis and CDD 3c cases with recurrent complicated diverticular disease [15]. The conversion rate from laparoscopic to open surgery in the patient subgroup classified as CCD 3c was significantly higher in the early elective surgical group. This patient subgroup is characterized by chronic symptomatic diverticular disease and, at the same time, presence of chronic inflammation-related sequelae such as fistulas and conglomerate masses of the pelvis. This finding suggests that these patients would benefit from a delayed-elective timing of surgery as they would not have experienced the drawbacks of open surgery. A further noteworthy aspect of the significantly different conversion rate within the CCD 3c patient subgroup is the fact that this classification of diverticular disease is the only one that further discriminates patients with chronic diverticular disease and substratifies them in groups where this difference can be detected by diagnostic imaging. Data that evaluate the German CDD classification is scarce. A recent study published by Lauscher et al. [16] demonstrated that subclassification of acute complicated stages into type 2a and 2b is reasonable as type 2b cases are more frequently operated at index hospitalization or during the early elective period with a longer associated hospital stay. In addition, Lauscher and co-workers [16] found that cases with CDD type 3c seem to have a more eventful postoperative course when compared to the CDD type 3b subgroup with uncomplicated recurrent diverticulitis. We now provide novel data that demonstrate the severity of CDD type 3c intraoperatively, as shown by the significantly higher conversion rate, underlining the challenging nature of surgery in the early elective setting. Existing evidence support that laparoscopy should be the surgical approach of choice when a one-staged colon resection is planned, as it results in superior short-term and comparable long-term outcome. This is supported by at least two randomized trials [19, 20]. Conversely, total health care costs of laparoscopic vs. open surgery were comparable. Although the indication of surgery in sigmoid diverticular disease depends on the disease stage and course [3], the ideal timing of sigmoid resection however remains unclear [6, 7, 11]. This question has far-reaching consequences as both early and delayed surgical approaches in complicated acute or recurrent attacks could potentially carry the risk of an unfavorable outcome after surgery. Reviewing the current literature, only a handful and mostly retrospective observational studies subjecting the ideal timing of resection in sigmoid diverticulitis and its impact on perioperative morbidity and mortality exist [10, 21–28]. While most studies show significantly lower conversion and complication rates in the delayed group [10, 22, 24, 27], some authors favor early resection because of similar morbidity and conversion rates as well as the potential risk of a complicated relapse during the waiting period in the delayed group necessitating urgent surgery [21, 25, 26]. Käser et al. [29] demonstrated that in patients with recurrent diverticular disease surgical management yielded a considerable positive symptom control which subsequently might display a higher patient preference towards early surgery in this group of patients. However, this should be weighed against the potential risks of a complicated operative course in early-resected patients. In our cohort, the conversion rate was significantly higher in the early group in comparison to the delayed group (26.1% vs. 6.7%). This observation is in line with other studies [10, 22, 24, 27]. However, the rate of overall postoperative complications including anastomotic insufficiency and the median length of hospital stay did not differ between the groups. Factors influencing hospital stay after colon resection have been identified in the literature [30, 31]. Of note, none of these studies focused specifically on laparoscopic sigmoidectomy in diverticular disease. We depicted these factors in elective sigmoid resection in our nomogram model. This tool helps clinicians as well as administrative hospital staff to precisely forecast hospital stay based on perioperative parameters and the operative course and therefore includes the results in their future economic considerations.

Regarding pathological examination of the harvested specimen, two studies show significant residual inflammation in patients with an early resection [22, 27]. In the study by Reissfelder et al., 66% of the conversions were necessary due to ongoing inflammatory processes [10]. These findings are similar to our observations indicating inflammatory adhesions as the most frequent cause of conversion in the early elective group. Indeed, three factors associated with a significantly elevated risk of conversion have been recently identified: surgical expertise, complicated sigmoid diverticulitis with stenosis or fistula, and severe inflammation on histological examination [32].

Our study has some limitations given its retrospective nature. We analyzed a small sample size over a relatively long study period. Nonrandomized patient selection for either early elective or delayed strategy and the varying experience of the performing surgeons are further biased in interpreting our data. Therefore, randomized controlled trials with unified classification and treatment protocols are needed to address optimal surgical timing in sigmoid diverticulitis and incorporate the results in the future national and international practice guidelines.

## Conclusion

Surgical treatment of colonic diverticular disease is stage and time dependent. Our proposed nomogram model provides a helpful clinical tool to identify and stratify patients with an increased risk of intraoperative conversion based on clinical parameters. Accordingly, the optimal timing of laparoscopic sigmoid resection should be adapted to the CDD. While early elective laparoscopic sigmoidectomy can be safely performed in acute uncomplicated diverticular disease stages, a higher risk of conversion to laparotomy in cases with acute or recurrent diverticulitis complicated by fistula, stenosis, large abscess, or stricture formation (CDD type 2b and 3c) justifies the delayed approach in the inflammation free episode 6 weeks after the index attack, when clinically possible. Nevertheless, the decision for an elective resection should still be made dependent on the clinical constellation. Therefore, a close follow-up during the waiting period is of particular importance in these patients, in order to recognize early recurrences during the waiting period and to avoid serious complications.

## Supplementary Information

Below is the link to the electronic supplementary material.

Supplementary file1 (DOCX 267 KB)
