# Supplementary material for: Early elective versus elective sigmoid resection in diverticular disease: not only timing matters—a single institutional retrospective review of 133 patients
Source: Langenbecks Arch Surg. 2022 Feb 22;407(4):1613–23. doi: 10.1007/s00423-022-02464-1 (PMC9283160; doi:10.1007/s00423-022-02464-1)
Supplement: Supplementary file 1 — Supplementary file1 (DOCX 267 KB) [file 423_2022_2464_MOESM1_ESM.docx]

**Supplementary Information**

**Early elective versus elective sigmoid resection in diverticular disease: Not only timing matters. A single institutional retrospective review of 133 patients**

Sascha Vaghiri, David Mario Jagalla, Dimitrios Prassas, Wolfram Trudo Knoefel, Andreas Krieg

Department of Surgery (A), Heinrich-Heine-University and University Hospital Duesseldorf, Duesseldorf, Germany

**Langenbeck's Archives of Surgery**

Corresponding Authors: Andreas Krieg, MD and Wolfram Trudo Knoefel, MD, FACS, Department of Surgery (A), Heinrich-Heine-University and University Hospital Duesseldorf, Moorenstr. 5, Bldg. 12.46, 40225 Duesseldorf, Germany; Email: andreas.krieg@med.uni-duesseldorf.de and knoefel@med.uni-duesseldorf.de

**Suppl. Fig. 1 CDD type distribution in patients (n = 133) undergoing sigmoid resection for diverticular disease**

**
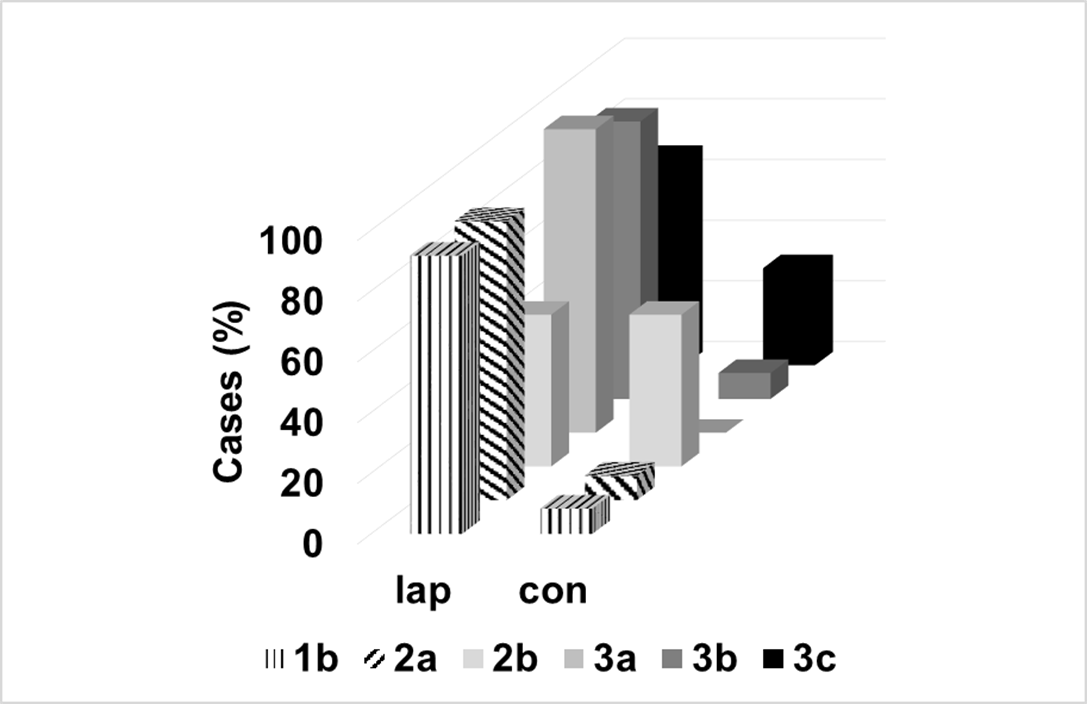
**


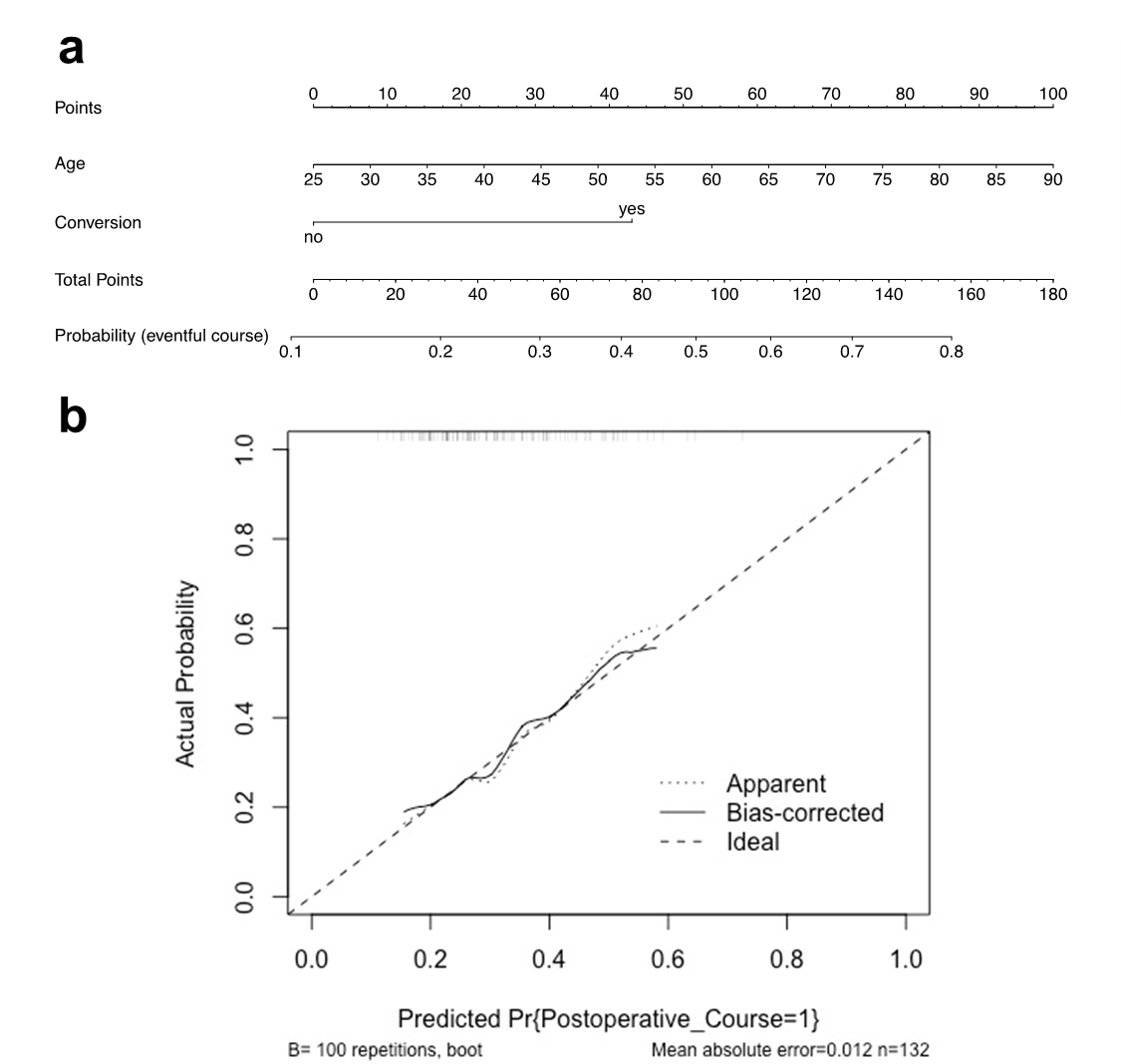
**Suppl. Fig. 2 Nomogram for predicting the probability of an eventful postoperative course after intended laparoscopic sigmoid resection.** Nomogram **(a)** built from a multivariate regression demonstrated a well fitted model in the calibration curve **(b)**
